# Correction: Classifying California’s stream thermal regimes for cold-water conservation

**DOI:** 10.1371/journal.pone.0269293

**Published:** 2022-05-26

**Authors:** Ann D. Willis, Ryan A. Peek, Andrew L. Rypel

This article [1] contains an error in the classification of one of the study sites. The NFA (North Fork American) site was classified as “unregulated” due to a code error. The correct classification is “regulated”.

In the Results section, there is an error in the fourth sentence of the ninth paragraph. The correct sentence is: Above California’s Central Valley rim dams, thermal regimes were mainly variable cool.

In the second subsection of the Discussion, there is an error in the fourth sentence of the first paragraph. The correct sentence is: Variable cool regimes occurred mainly in unregulated reaches, had more variable annual patterns (i.e., warmer annual maximums and cooler minimums), and had more predictable annual means, maximums, and day of annual maximum than stable cool regimes in regulated reaches. As a result of this variability, the sine model was a poorer fit for unregulated sites compared to regulated sites.

The following additional information is provided:

After the article [1] was published, concerns were raised about the importance of the study site elevation data. The concerns have been evaluated by the journal staff and a member of the Editorial Board. In light of this assessment, the journal determined that the article is scientifically sound and meets *PLOS ONE*’s publication criteria.

The authors provide the elevation data for all study sites in S3 Table below.

## Supporting information

S3 Table Site descriptors elevation data. This file includes elevation data for all study sites.
(CSV)
