# Supplementary material for: Classifying California’s stream thermal regimes for cold-water conservation
Source: PLoS One. 2021 Aug 20;16(8):e0256286. doi: 10.1371/journal.pone.0256286 (PMC8378740; doi:10.1371/journal.pone.0256286)
Supplement: Attachment — Submitted filename: Response_to_Reviewers.docx [file pone.0256286.s004.docx]

***Reviewer #1:*** *Manuscript Number: PONE-D-20-40523R1
Manuscript Title: Classifying California's stream thermal regimes for cold-water conservation

I reread the revised manuscript and think that the author’s have done a generally good job responding to concerns raised in my original review. If the author’s can address several additional items listed below, I think the paper could be made acceptable for publication on PONE. Most of the items are relatively minor, with the exception of revising the discussion section as noted below.

1. The Abstract needs revision. I’d suggest adding a few sentences between lines 25 and 26 to describe the dataset and analytical procedures. Otherwise, the conclusion statements starting on line 26 have no foundation.*

Thank you for the comment. We have revised the abstract to describe the dataset and general analytical procedures. We revised the abstract to include the following:

“We used publicly available, long-term (> 8 years) stream temperature data from 77 sites across California to model their thermal regimes, calculate three temperature metrics, and use the metrics to classify each regime with an agglomerative nesting algorithm. Then, we assessed the variation in each class and considered underlying physical or anthropogenic factors that could explain differences between classes. Finally, we considered how different classes might fit existing criteria for cool- or cold-water thermal regimes, and how those differences complicate efforts to manage stream temperature through regulation.” *2. Line 87. Sentence starts awkwardly, “Other, more data and computationally…” and could use revision.*

Thank you for the suggestion. We have revised the sentence to read as follows:

“Other approaches that require less data (< 5 years) or are computationally efficient bring considerable uncertainty in the results (10, 45, 46).” *3. Line 91 states “This study develops a classification framework…for rapid identification of stream reaches likely to sustain cool- and cold-water regimes.” The phrase “likely to sustain” implies to me that a temporal trend analysis will be done such that reaches which will remain cold in the future are being identified, despite climate change or other factors that may cause warming. I’d slightly rephrase this by deleting “likely to…” from the sentence since the analysis of regimes here is based on classifying discriminating characteristics for an eight-year snapshot of time.*

Thank you for the comment (and suggestion for further analysis!). We have revised the sentence to replace “likely to sustain viable” with “with”. *4. Line 128. Do the a, b, and n coefficients correspond to the mean, amplitude, and phase? This isn’t clear from the text or the associated figure.*
Thank you for the observation. Yes, coefficients a, b, and no correspond to annual mean, annual amplitude, and phase. We have clarified this in the manuscript with the following:

“…where *Tw* is water temperature, *n* is the day of water year, and *a*, *b,* and *n_o_* are coefficients that correspond to annual mean, annual amplitude, and phase (Fig 1). Coefficients *a*, *b,* and *n_o_* were optimized using least square regression.”

*5. Lines 187-188. This sentence belongs in the methods section. The standard name for this type of graph is an ordination plot I believe.*

Thank you for the suggestion. We removed those lines from the results, and revised the methods (line 160) to read: “We visualized the distribution of the clusters with an ordination plot of the first two principal components from the analysis, grouped by cluster.” *6. Line 193. If PC2 is most strongly correlated with phase, and the stable cold category of stream reaches a peak earlier than all the other classes (Fig 4c), why does this class plot intermediately along the PC2 axis (Fig 3) rather than at one of the extremes?*

Thank you for the question. While PC2 is correlated with phase, but is not completely driven by phase; thus we would not expect to see the class plot entirely at one of the extremes. Nevertheless, when we look at the position of each member in that group on the plot, they fall strongly along the PC2 axis, but in opposite directions. Thus, the centroid is located along the PC2 zero axis, but the members themselves illustrate a more extreme relationship. *Discussion section*

General comment: as we have completed a major revision of the discussion, we have not listed every instance of revised language as much of the feedback has been incorporated throughout the discussion. We have provided examples of lines where we specifically incorporated comments into our revisions.

*1. One limitation of the Maheu three parameter approach is that it ignores short-term variability (e.g., daily cycles and weekly variation) because it’s smoothing the annual cycle with a sine wave fit. In Isaak et al.’s 2020 classification of western U.S. stream thermal regimes based on dozens of metrics (reference 60 cited by the authors), that short-term variability was the primary determinant of PC2 (as was also the case in Rivers-Moore et al. 2013 multi-metric classification of South African streams; reference 46 cited by the authors) and others studying thermal regimes, as described in the discussion section of the Isaak paper, have argued that short-term thermal variation has particular ecological importance. In the discussion section of the present manuscript, it would be useful for the authors to elaborate on potential tradeoffs associated with using different metric sets for regime description and classification.*

Our method can be used as a rough cut to identify areas where conservation investment should be prioritized, particularly where desirable thermal regimes are independent of regulation. Once those areas have been identified, additional metrics such as those described in Isaak et al. and Rivers-Moore et al. can be used to understand the key metrics whose short-term variability has particular ecological importance, and that can be supported through process-based thermal regime management. We have added language to our discussion to specifically identify these limitations of our study (example, lines 334-339). We similarly address some of the comments regarding the range of metrics that have been developed, availability of long-term datasets, and interpolation methods (comments 2 & 3 below). We have condensed our discussion of these issues in the discussion section to focus more on the strengths of the findings, as per comment #4 below. *2. Lines 335-338. There are numerous papers that have already developed metrics to describe and explore stream thermal regimes based on frequency, rate of change, duration, magnitude, etc. some of which should be cited here (e.g., Steel et al. 2017 (reference 8 cited by authors in earlier context); Rivers-Moore et al. 2013 (reference 46 cited by authors in earlier context).*

Thank you for the comment. We have added language to the discussion to address this issue, cited the recommended studies (example, lines 334-339), and eliminated the speculative language around more research needed in this area from our discussion. *3. Paragraph lines 339-349. I think this paragraph needs significant revision because the potential already exists to mine information from a much larger database than the USGS & CDEC gage datasets that form the basis of the author’s analysis. The publicly available NorWeST database (*[*https://www.fs.fed.us/rm/boise/AWAE/projects/NorWeST.html*](https://www.fs.fed.us/rm/boise/AWAE/projects/NorWeST.html)*) contains stream temperature records for 3,681 unique sites in California as part of a much larger west-wide database. The dataset was published by Isaak et al. 2017 (Water Resources Research 53:9181-9205). Granted, most of the NorWeST records consist of short summer-only monitoring records but many do not and the records are easily sortable to extract those with more comprehensive records for regime analysis.

Also relevant to this paragraph of the discussion is the utility of modern imputation techniques for filling gaps in temperature records. In my experience, these work remarkably well with stream temperature records at both regulated and unregulated sites due to the strong temporal synchrony among sites, especially when the sites are part of dense monitoring networks as is the case here. The recent paper by Johnson et al. 2021 (Heed the data gap: guidelines for using incomplete datasets in annual stream temperature analyses. Ecological Indicators 122:107229) highlights the application of the imputation techniques developed by Josse et al. (2012. Handling missing values in exploratory multivariate data analysis methods, Journal of the Société Francaise de Statistique, 153, 79–99; and Josse and Husson 2016. MissMDA: a package for handling missing values in multivariate data analysis, Journal of Statistical Software 70: 1–31) to stream temperature records.*

Thank you for the comments. Regarding the observation of data available from NorWeST, we admit that we considered using this resource, but two issues persuaded us to limit our data queries to USGS and CDEC. First, our general impression from previous exploration with this database was that the majority of temperature records were short-term, summer-only monitoring. Second, the workflow of finding the longer-term records from NorWeST was not intuitive to us. Admittedly, this may be something that becomes less of an issue as we continue to explore and gain experience with this database. But at the time of this analysis, our lack of familiarity and previous experience with the NorWeST database prevented us from fully exploring its potential to supplement our study sites. Regardless, we have noted the database as a potential source for other studies in our manuscript (example: lines 362-365), and look forward to working more with it. Thank you for the suggestion and encouragement.

We have added language to our discussion to address the potential of filling datagaps using the methods identified in the suggested citations (example: lines 360-362). Thank you very much for the recommended sources.

*4. The discussion section as a whole at 11 pages is quite long compared to the overall 25 pages of text. I’d recommend looking for opportunities to streamline so that the strengths of the paper are highlighted while more speculative elements of the discussion are shortened or eliminated.*

Thank you for the comment. Characterizing some of the discussion as speculative was an incredibly helpful way of framing areas that crowded the stronger areas, and helped us quickly evaluate whether a statement or paragraph should be shortened or eliminated. We have made a major revision to our discussion to focus on the main areas of insight related to the results, and removed the more speculative observations. The revised discussion focuses on the definition of cold when referring to thermal regimes and the implications of alternative methods, and the role of dams in replicating/managing these regimes. We removed several paragraphs from the beginning of the discussion, which were mainly preamble or included statements that were repeated and explored in the context of the results later in that section. We also shorted our discussion of the methods, and included those points in relation to the other discussion topics. The remaining discussion allowed us to focus on the stronger elements of the paper, and retain additional discussion from the previous revision that expanded on dam regulation.

***Reviewer #2:*** *Thank you for revising your manuscript based on the comments. The revisions have made the manuscript clearer and more robust. In general, specific responses to the reviewer comments and associated revisions in the manuscript seem satisfactory. I do have a few minor comments to those below. All in all, this manuscript is an important contribution to the field of river temperature research and suitable for publication in PlosOne.

Minor comments:

Comment on Your response to Reviewer 2’ comment on Line 46-51:
Your explanation to the comment clarifies the rationale behind focusing on regulated reaches and the inclusion (or exclusion) of other factors. However, this rationale does not come across as clearly in the manuscript. I suggest to make this rationale more explicit in the introduction. The rationale in the introduction should also mention the novelty or research gaps that you are addressing (such as including in the paragraph starting line 91).*

Thank you for your comment and advice about the clarity of our explanation in our first response to reviewer comments. We have revised the introduction to include the clearer language in our earlier response and identify the research gaps we are addressing.  *Comment on Your response to Reviewer 2’ comment on Line 107:
Your response clarifies how you dealt with missing values. Although you have included a clarificatory line in the methods pertaining to this, I think it should be mentioned explicitly that data gaps/missing values were used as it as and not filled.*

Thank you for the suggestion. We have added the following line to the methods: “Any data gaps or missing values were not filled; the data was used as is.” *Lines 220-259: Including a table showing different thermal classes and their characteristics (mean, max, min, n, CV, DOWY etc) would be useful for better comprehension and for reducing the amount of text in these paras.*

Thank you for the suggestion. We have added a table to the main manuscript summarizing the number of members (n); average annual maximum and mean water temperatures; and day of annual maximum. We have also revised the text to remove some instances where we explicitly catalog the results, with the exception of statements where the results were necessary for clarity.

*Figure 4: Please also include the ‘n’ for each class within in the figure/legend/figure title.*

Thank you for the suggestion. The caption clarifies which portion of the figure indicates the ‘n’ for each class with the following language: “The number of members for each class (n) is as follows: stable warm (n = 1), variable warm (n = 30), variable cool (n = 12), stable cool (n = 32), stable cold (n = 2).” *There are still plenty of grammatical errors in the manuscript. Please correct them. Correcting some typos below:

Line 27: Groundwater streams are not a class of thermal regimes. They may exhibit a certain class of thermal regimes.
Line 34: worth “the” investment
Line 38: Replace “whereas” with “while”
Line 82: explore”d”
Line 146: "example" instead of examples
Line 151: Principal Components Analysis (PCA)
Line 395: “Some of these differences are” instead of “Some of this difference is”
Line 401: “importance of” instead of “important of”
Line 552: review”ers”*

Thank you for your careful reading. We have corrected the errors and typos that were listed, as well as others found during our revision.
